# Can metronomic maintenance with weekly vinblastine prevent early relapse/progression after bevacizumab–irinotecan in children with low‐grade glioma?

**DOI:** 10.1002/cam4.699

**Published:** 2016-03-31

**Authors:** Marie Amélie Heng, Laetitia Padovani, Philippe Dory‐Lautrec, Jean Claude Gentet, Arnaud Verschuur, Eddy Pasquier, Dominique Figarella‐Branger, Didier Scavarda, Nicolas André

**Affiliations:** ^1^ Service d'hématologie et Oncologie PédiatriqueCentre Hospitalo‐Universitaire Timone Enfants, AP‐HMMarseilleFrance; ^2^ Service de RadiothérapieCentre Hospitalo‐Universitaire Timone EnfantsAP‐HMMarseilleFrance; ^3^ Service de RadiologieCentre Hospitalo‐Universitaire Timone EnfantsAP‐HMMarseilleFrance; ^4^ Metronomics Global Health InitiativeMarseilleFrance; ^5^ Aix‐Marseille UniversitéINSERMCRO2 UMR_S 911Marseille13385France; ^6^ Service d'AnatomopathologieCentre Hospitalo‐Universitaire Timone EnfantsAP‐HMMarseilleFrance; ^7^ Service Neurochirurgie PédiatriqueCentre Hospitalo‐Universitaire Timone EnfantsAP‐HMMarseilleFrance

**Keywords:** Angiogenesis, cancer, children, low‐grade glioma, malignancies metronomic chemotherapy

## Abstract

The association of bevacizumab and irinotecan has been shown to display a quick efficacy in low‐grade glioma (LGG), but most patients relapse within months after cessation of therapy. From October 2012 to March 2014, four patients have been treated with irinotecan–bevacizumab followed by a metronomic maintenance with weekly vinblastine to try to prevent relapses. After a median follow‐up of 23 months after the end of the bevacizumab–irinotecan induction, no patient relapsed. These observations suggest that maintenance chemotherapy with weekly vinblastine after an induction by irinotecan–bevacizumab can improve progression‐free survival in children with LGG.

## Introduction

Low‐grade glioma (LGG) represents a third of primary central nervous system tumors in children, making it the most common brain tumor in childhood 1. Most patients with LGG have a prolonged survival, and for them LGG can be regarded as a chronic disease 2. Consequently, it is crucial to find treatments with less acute and long‐term toxicities. Previous studies have reported that a treatment with the association of bevacizumab with irinotecan could quickly improve symptoms 3, 4, 5, 6, which is highly relevant in case of visual impairment. However, most patients relapse within a median of 5 months after treatment cessation 4, 5. To prevent early relapses, we proposed a maintenance protocol with metronomic weekly vinblastine following irinotecan–bevacizumab induction therapy. The choice of vinblastine was based on evidence of antitumor activity of *Vinca* alkaloids in pediatric LGG 7, 8, 9, 10 and their good safety profile. Among anti‐ tubulin agents, vinblastine has been demonstrated to be an active drug even when used as a second line or more treatment 9. Lastly, vinblastine does not display auditory toxicity that is associated with carboplatin and which should be avoided in children with visual impairment. We report here a preliminary experience in four patients.

## Patients and Methods

### Patients

This is a retrospective analysis of four consecutive patients aged less than 18 years of age, treated with the association of bevacizumab–irinotecan as a first line treatment or for relapse of hypothalamo‐chiasmatic LGG from October 2012 to March 2014. Details about the patients, their underlying disease, and treatments are given in Table 1.

**Table 1:** Patients' characteristics and treatment

Patient ID	Gender	NF1	Age treated	Primary location	Metastasis	Pathology/Braf mutation	Previous therapies	Clinical status at treatment	Treatment response	FU
1	M	+	7	Visual pathway; Thalamus	No	Not done	None	Visual impairement	PR	16 months
2	F	−	13	Visual pathway	No	Pilocytic astrocytomaNo mutation Braf	None	Visual impairement	SD	16 months
3	F	−	16	Visual pathway	No	Not done	None	Visual impairement	SD	23 months
4	F	−	14	Posterior fossa; Pineal region	Yes (lepto‐meningeal)	Pilocytic astrocytomaMutation Braf.	(1) BB‐SFOP chemotherapy^a^(2) Surgery(3) Temozolomide(4) Carboplatinum‐Vincristine(5) Fluvastatin‐Celecoxib(6) Irinotecan‐bevacizumab (×2)(7) Metro‐SFCE01^b^	Visual impairementAtaxia Paraplegia	SD	5 months

NF, neurofibromatosis type 1; PR, partial response; SD, stable disease; FU, follow‐up.

a BBSFOP chemotherapy protocol consists in 3 week's cycles with alternating cyclophosphamide‐ vincristine; cisplatin‐etoposide; carboplatin‐procarbazine.

b Metro‐SFCE01 metronomic chemotherapy protocol consists ins: celecoxib‐vinblastine with alternating cyclophosphamide and methotrexate.

### Treatment

Patients were treated with the association of bevacizumab (10 mg/kg) and irinotecan (125 mg/m^2^) on day 1 and 15 of 2 weeks cycles 3, 4, during 6 months or until maximization of radiological or clinical benefit. Vinblastine was administered at the dose of 6 mg/m^2^ per week 8 and decreased to 3 mg/m^2^ in case of hematological toxicity. The planned total duration was 18 months. Patient no. 4 received oral vinorelbine (60 mg/m^2^) 10 instead of vinblastine since she progressed when receiving vinblastine with concomitant methotrexate, celecoxib, and cyclophosphamide as part of a phase II trial during the previous line of treatment 11.

### Evaluation

Cerebral and medullar MRI assessments were performed every 3 months. Responses were evaluated according to the RANO criteria.

## Results

Overall, treatment lasted 15 months (range: 13–24 months). The mean duration of induction was 9 months (7, 8, 10, and 12 months, respectively). The mean duration of maintenance with vinblastine was 7.5 months (8, 6, 3, and 12 months, respectively). Clinical improvement was noted in all patients, including vision improvement (*n* = 3) and ataxia (*n* = 1). In addition, after 3 months of treatment, one patient achieved objective response, and 3 patients had a stable disease, which were confirmed on following MRI. One patient who had stable disease while receiving the bevacizumab–irinotecan induction, responded to weekly vinblastine. The other 3 patients had a stable disease following vinblastine maintenance. No further visual improvement was noted during the maintenance phase. Most importantly, with a median follow‐up of 15 months (range: 5–23 months) after completion of maintenance therapy, no progression was noted.

Overall treatment was well tolerated. Toxicities observed during the irinotecan–bevacizumab was mild. No renal toxicity or hypertension was observed nor wound healing defect/delay. One patient (patient no. 3) had to stop irinotecan after 3 months because of grade III vomiting and nausea and grade II abdominal pain. The same patient also needed to stop vinblastine because of sustained grade II vomiting/nausea during maintenance therapy. For one patient (patients no. 1) vinblastine dose had to be reduced from 6 to 3 mg/m^2^ because of hematologic toxicity (grade III neutropenia).

## Discussion

Achieving to maintain sustained stable disease in pediatric patient with LGG, while limiting both acute and long‐term toxicities is challenging 2, 3, 4, 5, 6, 7, 8, 9, 10. Recently, the combination of bevacizumab and irinotecan has been reported to produce rapid tumor response in some children with recurrent LGG 3, 4, 5, 6, but patients frequently relapse shortly after stopping treatment 4. Indeed, Hwang and co‐workers reported that 13 out of 14 patients progressed after stopping bevacizumab at a median time of 5 months 4. In this report, a maintenance therapy with metronomic weekly vinblastine was added after induction with bevacizumab–irinotecan to prevent early relapses. Noteworthy, the visual improvement obtained during the induction phase was maintained during the vinblastine metronomic treatment. Overall, this approach lead to the stability or improvement of symptoms in the four patients with a median follow‐up of 16 months after completion of treatment, and 23 months after the end of induction therapy with bevacizumab–irinotecan. Although the number of patients and follow‐up are limited, the results we report herein compare favorably with the different studies investigating the use of bevacizumab–irinotecan in patients with LGG 4.

This treatment was well tolerated, which is in line with previous reports of good safety profile of bevacizumab–irinotecan 3, 4, 5, 6 and vinblastine 8, 9 in children with LGG. In this study, irinotecan had to be stopped in one patient after 7 months of treatment because of digestive toxicity, and single agent bevacizumab was continued for 3 more months. Vinblastine maintenance had to be stopped prematurely in this patient due to general bad tolerance of the treatment. Vinblastine dose reduction was also necessary in another patient because of hematologic toxicity. Side effects were quickly reversible after treatment cessation and tolerance was improved after dose reduction.

The maintenance phase relies on IV injections because of the lack of oral formulation of vinblastine. Vinorelbine has been recently reported to be an active drug for the treatment of relapsing/refractory LGG 10. It is orally available, thus paving the way for an oral maintenance metronomic regimen. This approach would eventually reduce the cost of treatment and the number of stays in hospital. In this study, one patient was treated with oral vinorelbine for 12 months. Treatment was well tolerated.

Here, the maintenance regimen with weekly vinblastine is based on frequent administration of chemotherapy at relatively low dose and can therefore be regarded as a metronomic treatment 12. This approach has already been reported to be active in LGG 13, 14. Acknowledging that maintenance therapy is metronomic brings new light on the mechanisms of action that can contribute to long‐term control of the disease 15. Metronomic chemotherapy has been reported to be antiangiogenic and to restore some level of antitumor immune response 12, thus potentially re‐inducing tumor dormancy, which can be beneficial in patients with LGG.

We report here, preliminary observation of the potential clinical benefit of adding metronomic maintenance with vinblastine after initial treatment with an association of bevacizumab–irinotecan to try to prevent early relapse. Larger randomized studies aiming at demonstrating the value of a vinblastine‐based maintenance regimen in patients with LGG after irinotecan–bevacizumab induction are mandatory. Alternatively, using oral vinorelbine instead of vinblastine might contribute to improvement of patient's quality of life.

## Conflict of Interest

The authors have no conflict of interest to declare.
